# Association between nutritional status and socio-economic status among school children aged 9–17 years in a semi-urban area of Nepal

**DOI:** 10.1186/s41043-023-00392-4

**Published:** 2023-06-08

**Authors:** Sophie Amalie Hamann, Lene Thorup, Cecilie Blenstrup Patsche, Lena Hohwü, Vibeke Elisabeth Hjortdal, Bishal Gyawali, Dinesh Neupane, Per Kallestrup

**Affiliations:** 1 Department of Public Health, Center for Global Health (GloHAU), Aarhus University, Aarhus, Denmark; 2 Department of Cardiothoracic Surgery, Copenhagen University Hospital, Blegdamsvej 9, 2100 Copenhagen, Denmark; 3 The Research Center for Health and Welfare Technology, VIA University College Aarhus, Aarhus, Denmark; 4 Department of Public Health, Global Health Section, University of Copenhagen, Copenhagen, Denmark; 5 Department of International Health, Johns Hopkins University Bloomberg School of Public Health, Baltimore, USA; 6 Nepal Development Society, Bharatpur-05, Chitwan, Nepal

**Keywords:** Malnutrition, Child, Adolescent, Nepal, Stunting, MUAC

## Abstract

**Background:**

In many low-and middle-income countries (LMICs), childhood overweight is increasing, while underweight remains a problem. This study aimed to investigate the association between socio-economic status (SES) and nutritional status among Nepalese school children.

**Methods:**

This cross-sectional study used a multistage random cluster sampling method and included 868 students aged 9–17 years from both public and private schools located in a semi-urban area of Pokhara Metropolitan City, Nepal. SES was determined based on a self-reported questionnaire. Body weight and height were measured by health professionals and body mass index (BMI) was categorized based on the World Health Organization BMI-for-age cut-offs. The association between Lower and Upper SES and BMI was assessed using mixed-effects logistic regression model estimating the adjusted odds ratio (aOR) with a corresponding 95% confidence interval (CI) and compared to Middle SES.

**Results:**

The proportion of obesity, overweight, underweight, and stunting among school children was 4%, 12%, 7%, and 17%, respectively. More girls were overweight/obese compared with boys (20% vs. 13%). The mixed-effects logistic regression model showed that both participants from Lower SES households and Upper SES households had a higher tendency to be overweight compared to participants from Middle SES; aOR = 1.4; 95% CI 0.7–3.1 and aOR = 1.1; 95% CI 0.6–2.1, respectively. Furthermore, stunting and overweight occurred simultaneously.

**Conclusions:**

This study found that about one out of four children and adolescents in the study setting was malnourished. There was a tendency that both participants from Lower SES and Upper SES had higher odds of being overweight compared to participants from Middle SES. Furthermore, both stunting and overweight were present simultaneously in some individuals. This emphasizes the complexity and importance of awareness of childhood malnutrition in LMICs like Nepal.

## Background

Overnutrition is a global public health problem in both adults and children [1–3]. The prevalence of overweight and obesity among children in low-income and middle-income countries (LMICs) is currently accelerating [4–6]. In 2020, an estimated 38.9 million children worldwide under 5 years of age were overweight [7]. Childhood obesity increases the risk of adiposity in adulthood [8] which substantially increases the risk of non-communicable diseases such as cardiovascular diseases, hypertension, diabetes, and cancer [9–13].

In LMICs, higher socio-economic status (SES) is positively associated with overweight/obesity [14, 15]. It has been suggested that this association becomes weaker or even disappears as the overweight/obesity epidemic is progressing [16]. Thus, the association between SES and obesity is complex and some children in LMICs are overweight and stunted simultaneously [17], as part of a double burden of malnutrition [4, 18].

As a low-income country [9], Nepal faces great health challenges as a consequence of its ongoing urbanization and diet transition [19]. The rates of overweight/obesity have nearly tripled in Nepal since the 1980s [20]. In 2015, the prevalence of overweight and obesity among Nepalese children aged 2–19 years was 4.6% and 1.7% for boys and 3.5% and 1.1% for girls, respectively [20].

Knowledge about the relationship between SES and nutritional status among Nepalese school children has so far been limited and no previous research on this topic has been carried out in a semi-urban setting of Nepal. Thus, the aim of this study was twofold: (i) to explore the prevalence of obesity, overweight, underweight, and stunting among school children aged 9–17 years in a semi-urban area of Pokhara Metropolitan City of Nepal, and (ii) to examine whether SES of the household was associated with BMI among school children aged 9–17 years.

## Methods

### Study setting, design and participants

This cross-sectional study was conducted in a semi-urban area of the Pokhara Metropolitan City of Nepal. Data were collected from March to May 2018. Inclusion criteria were 9–17-year-old school children and attended class on the day of data collection. Exclusion criteria were: missing written consent, missing data for BMI or questionnaire, and any mental or psychological disorders.

Participants were randomly selected through a multistage cluster sampling method. Enrolment lists of all 60 schools in the Pokhara Metropolitan City were provided by Kaski District Education Office. Sixteen schools only offered education up to 5th grade and were thus excluded. Of the remaining 44 schools, two separate lists of public and private schools were generated (21 private and 23 public schools). Using a lottery method, 18 schools from the two lists were randomly selected (10 private and 8 public). Random selection was likewise performed for the inclusion of a minimum of 45 children from each school attending 5th to 9th grade. The school system in Nepal requires a passed exam to move to the next grade. This means that the age of the children in different grades is heterogeneous.

### Data collection

Data were collected by a fieldworker who had public health degree and was trained by the principal investigator (PI). A questionnaire was initially prepared in English and then translated into Nepali (the national language). The students filled out the questionnaire and the fieldworker provided guidance when necessary. Subsequently, the field worker and PI measured height, weight and mid-upper arm circumference (MUAC).

### Self-administered questionnaire

The self-administered questionnaire included socio-demographic factors such as sex, date of birth, ethnicity, family type, maternal educational level, and occupation. The exposure variable, SES of the household, was calculated based on information about living conditions, household items and livestock adapted from Piryani et al*.* [21] which is based on questions from the Nepal Demographic and Health Survey [22]. SES was then classified into three categories according to the Equity Wealth Quintile Guide [23]: Lower SES, Middle SES and Upper SES. Terciles were used instead of quintiles due to the smaller sample size of this study compared to larger epidemiological surveys. To make the calculations and division of SES, Principal Component Analysis was used.

### Anthropometric measurement

Anthropometric measurements were performed according to World Health Organization (WHO) standards [24]: body weight was measured to the nearest 0.1 kg (kg) on a Báscula digital scale. Heavy clothes and shoes were removed beforehand. Height was measured in metres to the nearest 0.1 cm by a meter tape and without shoes. MUAC was measured on the left arm: the measurement was performed at the mid-point between the olecranon and acromion on a relaxed arm.

### Classification of variables

BMI was calculated as weight in kg divided by height squared in meters (kg/m2). The age of the child was based on the birthday of the child from the Nepali calendar and calculated into the age in years and months. The z-scores for BMI were determined and participants were divided into groups of obesity (≥ 2 standard deviations (SD)), overweight (≥ 1 SD), normal weight and underweight (< − 2 SD) based on age and sex according to WHO growth reference BMI-for-age scores for 5–19 years [25]. Children were considered stunted if sex-specific height-for-age z-scores were below minus two SD according to WHO guidelines [25].

### Pretesting

The pretesting of the questionnaire took place at a school not included in the sampling framework. Ten children in 9th grade (14–15 years) completed the questionnaire in 15 min. The pretesting showed that the children were not able to answer the question: “how many bigha/katha (the unit used to measure land in Nepal) of agricultural land does your family own?”. Hence, the question was excluded from the study.

### Sample size

Based on data from the Global Burden of Disease 2015 Obesity Collaborators [20], the estimated prevalence of overweight was 10%. Furthermore, a non-response rate of 20% was assumed. The sample size was calculated by multiplying the design effect (1.5) and the number of socio-economic strata (3) resulting in a sample size of 778.

### Statistical analyses

Data were entered in the REDCap database and analyzed using STATA statistical software version 15 (Stata Corporation, College Station, Texas, USA). Data were checked for linearity, normality, and collinearity before conducting regression analysis. Multivariate mixed-effects logistic regression analysis was used to calculate the adjusted odds ratio (aOR) with a corresponding 95% CI, as a measure of the association between SES of the household and BMI. Potential confounders considered were: age, age-squared, sex, school type, maternal literacy, maternal employment, family type, and ethnicity.

### Ethical considerations

Permission to conduct this study was obtained from the Ethical Review Board of Nepal Health Research Council (reg no: 538/2017). Consent to participate in the study was sought from the child and the parents/legal guardian. Written and oral information was given in Nepali to the children and school principals. Signed informed consent from both the participating child and parents/legal guardian, either written or by fingerprint, was obtained. The study abides by “The ethics of research related to healthcare in developing countries” by the Nuffield council on bioethics [26]. Children found to be either overweight, obese or underweight received a letter written in Nepali for their parents or legal guardian and were referred through the channels of local Female Community Health Volunteers to primary health care centers.

## Results

Based on the enrolment list, 1041 children were expected to be included in the study. Eighteen children attending the classes on the day of the visit did not appear on the list but were considered eligible for inclusion. A total of 147 children were excluded due to: absence from class (n = 145), mental retardation (n = 1), and rejected participation from parents/legal guardian (n = 1). Of the 912 students eligible for the study, 44 were excluded due to: missing written consent from parents/legal guardian (n = 42), missing age (n = 1), and age > 17 years (n = 1). The final study population included 868 children (response rate = 83%), of wich 428 were boys (49%) and 440 girls (51%). Descriptive characteristics of the study population are presented in Table 1.

**Table 1:** Descriptive characteristics of the study participants by SES (n = 868)

	Lower SES n = 292	Middle SES n = 241	Upper SES n = 335	Combined n = 868
Age in years mean ± SD	12.6 ± 1.5	12.6 ± 1.3	12.7 ± 1.3	12.7 ± 1.4
Sex, n (%)				
Girls	143 (49)	126 (52)	171 (51)	440 (51)
Boys	149 (51)	115 (48)	164 (49)	428 (49)
MUAC in cm mean ± SD	20.8 ± 2.6	20.8 ± 2.5	21.9 ± 2.9	21.2 ± 2.7
BMI-for-age groups, n (%)				
Underweight	16 (6)	20 (8)	26 (8)	62 (7)
Normal weight	227 (78)	188 (78)	246 (73)	661 (76)
Overweight	36 (12)	25 (10)	47 (14)	108 (12)
Obesity	13 (4)	8 (3)	16 (5)	37 (4)
Height-for-age, n (%)				
Stunted	61 (21)	41 (17)	43 (13)	145 (17)
Normal height-for-age	231 (79)	200 (83)	292 (87)	723 (83)
Ethnicity, n (%)				
Dalits	91 (31)	61 (25)	33 (10)	185 (21)
Disadvantaged Janajatis	94 (32)	61 (25)	76 (23)	231 (27)
Advantaged Jajanatis	25 (9)	25 (11)	65 (19)	115 (13)
Upper caste	68 (23)	89 (37)	154 (46)	311 (36)
Other/do not know	13 (5)	4 (2)	7 (2)	24 (3)
School type, n (%)				
Public	192 (66)	107 (44)	81 (24)	380 (44)
Private	100 (34)	134 (56)	254 (76)	488 (56)
Maternal education, n (%)				
Illiterate	35 (13)	6 (3)	11 (4)	52 (7)
Literate	237 (87)	208 (97)	298 (96)	743 (93)
No education	1 (0)	2 (1)	0 (0)	3 (0)
Primary to secondary level	156 (53)	150 (62)	196 (59)	502 (58)
Higher secondary to bachelor and above	15 (5)	16 (7)	75 (22)	106 (12)
Do not know	120 (41)	73 (30)	64 (19)	257 (30)
Maternal employment, n (%)				
Housewife	165 (57)	172 (72)	224 (67)	561 (65)
Working	126 (43)	68 (28)	110 (33)	304 (35)
Do not know	1 (0)	1 (1)	1 (0)	3 (0)

*BMI* Body mass index, *MUAC* Mid-upper arm circumference

Missing values: MUAC: 183, maternal literacy: 73

Of all participants, 17% (n = 145) were overweight/obese and 7% (n = 62) were underweight. The overweight/obese participants were almost evenly distributed across SES, but the prevalence was higher amongst girls (20%) than boys (13%). Similarly, the number of underweight were also evenly distributed across SES, but the prevalence of underweight was twice as high in boys (10%) than in girls (5%).

The association between SES and both underweight and overweight/obesity is shown in Table 2.

**Table 2:** Mixed-effects logistic regression analysis of the association between SES and underweight and overweight/obese showed in odds ratio (OR) with Middle SES as the reference (n = 868)

	Underweight		Overweight/Obese	
	Unadjusted OR (95% CI)	Adjusted OR* (95% CI)	Unadjusted OR (95% CI)	Adjusted OR* (95% CI)
Lower SES	1.3 (0.7–2.1)	1.4 (0.8–2.5)	1.5 (0.8–3.0)	1.4 (0.7–3.1)
Middle SES	1.0 (Reference)	1.0 (Reference)	1.0 (Reference)	1.0 (Reference)
Upper SES	13. (0.8–2.2)	1.3 (0.8–2.3)	1.0 (0.5–1.9)	1.1 (0.6–2.1)

*SES* Socio-economic status, *CI* Confidence interval, *OR* Odds ratio

*Adjusted for age, age-squared, sex, school type, maternal literacy, family type, ethnicity and maternal employment

Both participants from Lower SES households (aOR = 1.4; 95% CI 0.7–3.1) and participants from Upper SES households (aOR = 1.1; 95% CI 0.6–2.1) had higher odds of being overweight compared with participants from Middle SES households. The tendency was more pronounced for participants from Lower SES households.

Participants from Lower SES households had same odds (aOR = 1.4; 95% CI 0.8–2.5) of being underweight as participants from Upper SES households (aOR = 1.3; 95% CI 0.8–2.3). None of these OR are statistically significant.

**Fig. 1:**
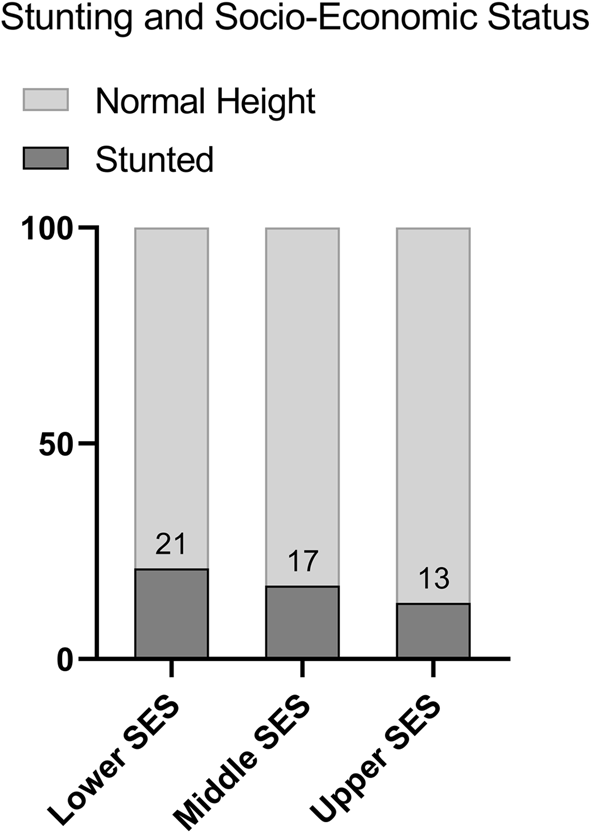
Prevalence of stunting across Socioeconomic Status. *SES* Socio-economic status

**Fig. 2:**
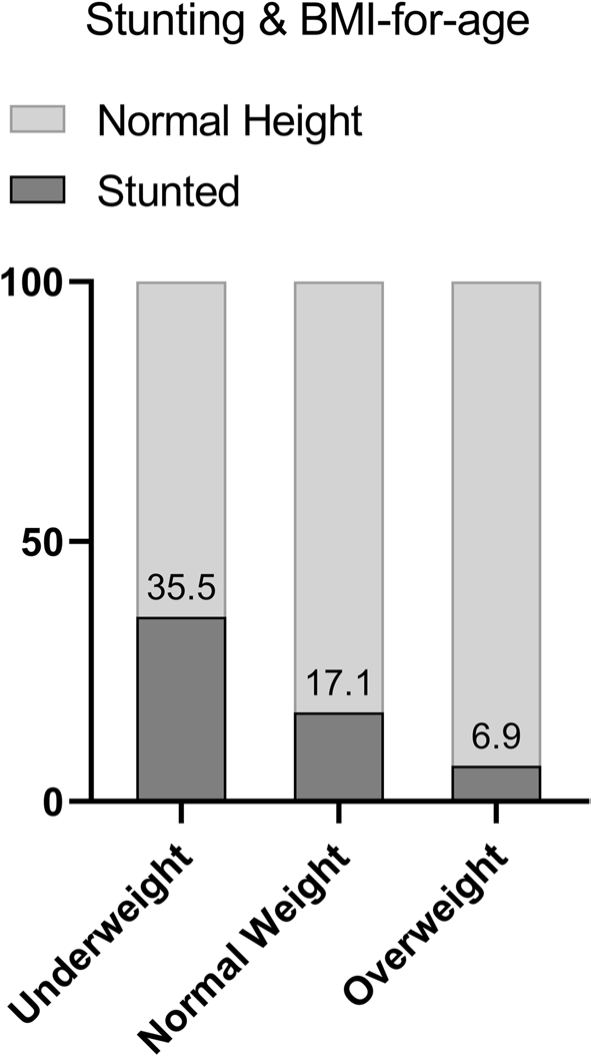
Prevalence of stunting across BMI groups, *BMI* Body mass index

In total, 145 (16.7%) participants were stunted. Stunting prevalence was inversely related to SES with 21% of Lower SES, 17% of Middle SES and 13% of Upper SES being stunted (Fig. 1). The same, more pronounced relationship was observed between stunting and BMI categories. In total, 35.5% of all underweight were stunted, declining to only 6.7% of all overweight (Fig. 2).

## Discussion

In this cross-sectional study, almost one out of four (24%) of 868 school participants aged 9–17 years in the semi-urban area of Pokhara Metropolitan city were malnourished (overweight/obese or underweight), with the majority being overweight/obese. Both participants from Lower SES and Upper SES had higher odds of being overweight compared to those from Middle SES. Though not statistically significant, malnutrition seemed to appear in both lower and upper SES of the society. These results should be interpreted with caution and points more towards a tendency than an association. The study also showed that girls were more likely to be overweight than boys. Similar studies have been carried out across Nepal [21, 27–29, 38], the results are summarized in Table 3.

**Table 3:** Overview of the literature

Author	Study size	Age, Years	Setting	Overweight/Obese	Underweight	Stunted
Piryani et al. 2016 [21]	360	16–19	Public & Private School	12.2%	–	–
Koirala et al. 2015 [27]	986	6–13	Private School	25.9%	10.3%	–
Stewart et al. 2013 [28]	4729	9–13 14–23	Rural	1% 5%	–	56.1% 41.2%
Mansur et al. 2015 [29]	438	4–16	Rural	–	10.05%	24.5%
Nepal National Micronutrient Status Survey, 2018 [38]	981 boys 1722 girls	10–19 10–19	Urban & Rural	6% 4.7%	28% (wasting) 17% (wasting)	43% 40%
Hamann et al. (current study)	868	9–17	Public & Private School	16.7%	7%	17%

The prevalence of overweight children in the study by Piryani et al. is in line with the findings of our study. The tendency that children from Upper SES had higher odds of being overweight found in our study is consistent with the findings by Koirala et al. and Piryani et al. However, neither of the two presented data on the association between children from lower groups of SES and overweight, hence we cannot compare this aspect of our study findings.

Additionally, we found a much lower proportion of underweight and stunting than Mansur et al. and Stewart et al. However, they included only children from the rural part of Nepal, whereas our study was conducted in a semi-urban area that includes both urban and rural areas. This may be due to a difference in lifestyle between rural and urban Nepal, which could have an impact on nutritional status in childhood [30]. This is however in contrast to the higher proportions of underweight and stunting also reported in the National Micronutrient Survey, but the report likewise concludes that there is significant geographical variation.

Stunting prevalence tended to decrease with higher BMI and SES. While not surprising to see the highest prevalence of stunted amongst the underweight, it is worth noting that there were also stunted, overweight adolescents. This could be a sign of the double burden of malnutrition eg. concurrent undernutrition and overweight/obesity [31].

Stunting develops almost exclusively during the first 1000 days from conception—that is during fetal life until the child reaches ~ 2 years of age [32]. Maternal undernutrition compromises fetal growth and increases the risk of the child becoming stunted [33].

While the relationship between growth patterns and body composition within life courses is very complex, there is a strong association between rapid weight gain within the first 1000 days of life and lean body mass, whereas rapid weight gain after this point is associated with adult fat mass. Especially those who were undernourished as infants but later in childhood experienced rapid weight gain seem to be at increased risk of adult overweight [32].

This information has to be seen in the light of the ongoing global epidemiologic changes which entail both nutritional, epidemiological and demographic transitions. Whereas these processes have taken place slowly over centuries in high-income countries, many LMICs have experienced an accelerated epidemiologic change over just decades. These rapid changes have caused intragenerational transformations leading to e.g. an overlap of underweight/stunting and later overweight in the same individual [34]. Consistently, data from the World Bank (2016) [35] showed that the GDP and GDP per capita are both steeply increasing in Nepal, and high-fat and animal-source foods are increasingly consumed among low-income groups in LMICs as country-level GDP increases [36].

These processes could be triggering what we are seeing in our study; that some of the children from the lower SES are both overweight and stunted. We speculate that they could have suffered from undernutrition during fetal development and early life making them stunted, and then, later on, experienced a diet transition and became overweight.

This is to our knowledge the only study that has examined an association between SES and BMI among school children in the semi-urban area of Nepal. The high number of participants is the main strength of this study. The outcome (BMI) was measured by the PI and trained research assistant. This decreases the risk of differential misclassification. Calculations on the SES of the household were based on household items instead of monthly income as a marker for SES. This was done as monthly income may vary by season if household members have short periods of employment. Thus, our measurement of SES increases the validity of our findings. The 147 excluded participants were from all three groups of SES and therefore selection problems have not likely biased the results.

We acknowledge that the study also had a number of limitations. Firstly, data collection by questionnaire may have led to information bias. It may be possible that participants from Lower SES overreported information about the household. This could lead to differential information bias and underestimation of the association between BMI and SES. Secondly, the associations could have been biased by residual confounders. However, it remains unknown whether this situation may weaken or strengthen the associations. Furthermore, this study represents a semiurban area of Nepal, and thus not representative of the entire country. This should be considered as a limitation and taken into consideration before applying in other areas or on a national scale. Finally, Rojroongwasinkul et al. calculated body length/height percentiles among 14,202 children aged 0–12 years in South-East-Asia [37]. When comparing their findings to WHO standard curves it was shown, that height percentiles were lower in almost all ages and percentiles in both sexes, indicating skewness to the left. The current study used WHO standard curves when determining stunting rates. The findings by Rojroongwasinkul et al*.* could be considered applicable to the present study, and if so, would have yielded a lower prevalence of stunted children.

## Conclusion

In children and adolescents from the semi-urban area of Nepal, one in four children were malnourished, with the majority being overweight/obese. This study found a tendency towards an association between both lower and upper SES and childhood overweight, suggesting nutritional challenges in both lower and upper parts of the society. Overweight/obesity in Upper SES is previously described whereas our finding of overweight/obesity in children from lower SES is new in this setting. Furthermore, both overweight and stunting were present in the same individual, but stunting prevalence was markedly higher in low SES. This could be part of a nutritional transition and increase in GDP occurring in Nepal. In summary, this study displays the complexity of childhood malnutrition in LMICs like Nepal, warranting broader attention to all aspects of malnutrition across all socioeconomic levels in society.

## Data Availability

The datasets used and/or analysed during the current study are available from the corresponding author on reasonable request.

## Abbreviations

BMI: Body mass index
CI: Confidence interval
GDP: Gross domestic product
kg: Kilogram
LMICs: Low- and middle-income countries
MUAC: Mid upper arm circumference
aOR: Adjusted odds ratio
PI: Principal investigator
SD: Standard deviations
SES: Socioeconomic status
WHO: World Health Organization
